# Molecular Stress Responses to Nano-Sized Zero-Valent Iron (nZVI) Particles in the Soil Bacterium *Pseudomonas stutzeri*

**DOI:** 10.1371/journal.pone.0089677

**Published:** 2014-02-25

**Authors:** Maria Ludovica Saccà, Carmen Fajardo, Montserrat Martinez-Gomariz, Gonzalo Costa, Mar Nande, Margarita Martin

**Affiliations:** 1 Facultad de Veterinaria, Universidad Complutense de Madrid, Madrid, Spain; 2 Moncloa Campus of International Excellence, Madrid, Spain; 3 Proteomic Facility, Universidad Complutense de Madrid, Madrid, Spain; Indian Institute of Toxicology Reserach, India

## Abstract

Nanotoxicological studies were performed *in vitro* using the common soil bacterium *Pseudomonas stutzeri* to assess the potentially toxic impact of commercial nano-sized zero-valent iron (nZVI) particles, which are currently used for environmental remediation projects. The phenotypic response of *P. stutzeri* to nZVI toxicity includes an initial insult to the cell wall, as evidenced by TEM micrographs. Transcriptional analyses using genes of particular relevance in cellular activity revealed that no significant changes occurred among the relative expression ratios of *nar*G, *nir*S, *pyk*A or *gyr*A following nZVI exposure; however, a significant increase in *kat*B expression was indicative of nZVI-induced oxidative stress in *P. stutzeri.* A proteomic approach identified two major defence mechanisms that occurred in response to nZVI exposure: a downregulation of membrane proteins and an upregulation of proteins involved in reducing intracellular oxidative stress. These biomarkers served as early indicators of nZVI response in this soil bacterium, and may provide relevant information for environmental hazard assessment.

## Introduction

Nanomaterials, which are characterised as materials containing particles smaller than 100 nm, are becoming increasingly important nanotechnology products and are the centre of intense research because of their unknown environmental risks and effects on living organisms. Among nanomaterials, nano-sized zero-valent iron (nZVI) particles represent a new generation of products used for environmental remediation strategies and are considered to be a valid option for treating contaminated soil and groundwater systems [1], [2]. In the USA, nZVI treatment is a well-accepted practice, whereas until recently, few applications have been carried out in Europe, a region that lacks regulations for nZVI environmental remediation practices. A paucity of research that assesses the health and environmental risks of nZVI treatment is currently impeding its commercialisation [3].

A variety of possible nZVI toxicity mechanisms toward bacteria have been investigated, including the disruption of cell membrane integrity, the interference with respiration, DNA or protein damage, and oxidative stress by the generation of reactive oxygen species (ROS) [4], [5]. High intracellular ROS concentrations can activate redox-sensitive pathways, which in turn can lead to the activation of a variety of scavenging enzymes [2], [6]. Regardless of the exact mechanism, nZVI toxicity on the microbial community is not well understood, although previous studies have indicated that toxicity seems to be dose- and species-dependent [1].

The identification and validation of new markers to assess nanomaterial toxicity are needed and “omic” tools would provide complementary information to the classical ecotoxicological tests that are currently available. Hence, the transcriptional changes of bacterial genes such as those encoding enzymes involved in the nitrogen biogeochemical cycle (e.g., nitrate reductase (*nar*G) and nitrite reductase (*nir*S)), DNA replication (e.g., gyrase (*gyr*A)), glycolysis (e.g., pyruvate kinase (*pyk*A)), and the cellular oxidative stress response (e.g., catalase (*kat*B)) can provide information about the impact of nanoparticles on environmental microorganisms.

Proteomics is also recognised as a valuable tool in ecotoxicology research; the term ‘ecotoxicoproteomics’ is used to define protein profiling techniques developed for the purpose of identifying new treatment-related markers that are capable of providing information about early responses to a toxicant [7].

A previous study on the impact of nZVI on soil microbial population, when used as an immobilization strategy in heavy metal contaminated soils [1] showed significant shifts in structure and composition of the soil bacterial population. Among the studied groups, the Gram positive *Firmicutes* and the Gram negative γ-*Proteobacteria* were the most dramatically affected.

In light of these findings, we focused on the impact of commercial nZVI on *Pseudomonas stutzeri* (belonging to γ-*Proteobacteria*) using *in vitro* analyses. *P. stutzeri* is a Gram negative denitrifying bacteria, widely distributed throughout the environment and considered a model species for environmental research. The phenotypic (growth, morphology) and molecular (differential gene and protein expression) responses of *P. stutzeri* following nZVI exposure were analysed to identify new stress response-specific biomarkers.

## Materials and Methods

### 1. Commercial Nanoparticles

Iron nanoparticles (NANOFER 25S) were commercially synthesised and supplied by NANO IRON s.r.o. (Rajhrad, Czech Republic) as an aqueous dispersion of stabilised nZVI (coated with sodium polyacrylic acid 3%), with an average particle size of <50 nm. The iron content was 70–90% and 10–30% iron oxides when produced. Additional details about the physical and chemical characteristics are available at www.nanoiron.cz. Nanoparticles were used immediately after reception.

### 2. Effect of nZVI on Bacterial Growth

The test bacterium, *Pseudomonas stutzeri* (ATCC14405) was purchased from DSMZ (www.dsmz.de). The bacterial culture was incubated in Luria-Bertani (LB) broth at 37°C until the exponential growth phase was reached. Cells were then pelleted and washed with a 9 g L^−1^ sodium chloride solution. The culture was resuspended in the same solution, supplemented with varying nZVI concentrations (1, 5, 10 g L^−1^) and incubated at 37°C for 0.2, 2, 24 and 48 h, in the dark. Cultures without nanoparticles were used as controls. The toxicity was evaluated by counting the number of colony forming units (CFU) on LB agar plates produced by treated bacteria compared to untreated controls. All of the treatments were prepared in duplicate, and each set of experiments was repeated three times to ensure reproducibility. The results from bacteria propagation were statistically analysed using an ANOVA test (p<0.05).

### 3. Electron Microscopy Analysis

The morphology of bacterial strains, in both untreated and those treated by 3 h of incubation with 5 g L^−1^ of nZVI was examined by transmission electron microscopy (TEM) (Centro de Microscopía y Citometría, UCM facility), using standard procedures for fixing and embedding sensitive biological samples [8].

### 4. Transcriptional Response to nZVI Exposure

The expression of *narG* and *nirS* genes was analysed using the primer sets previously described [1]. Three sets of primers were custom-synthesised and developed by PrimerDesign Ltd. (Southampton, UK) to specifically amplify *gyrA*, *pykA*, and *katB* genes in the *P. stutzeri* strain (Table 1). Primer design was carried out using Primer Select, megAlign (DNAStart, Inc. 1999, 5th Edition, Wisconsin) and the National Centre for Biotechnology Information Blast Search Program (http://www.ncbi.nlm.nih.gov/).

**Table 1:** Characteristics of the set of RT-qPCR primers used in this study.

Gene	Primer sequences 5′-3′	Specificity	Reference
*nar*G	F-TACTGTGCGGGCAGGAAGAAACTG	*Eubacteria*	[1]
	R-CGTAGAAGAAGCTGGTGCTGTT		
*nir*S	F-CCTAYTGGCCGGCRCART	*Eubacteria*	[1]
	R-GCCGCCGTCRTGVAGGAA		
*pyk*A	F-TGTACACCGCCAACCACTT	*P. stutzeri*	This study
	R-GGATGCGCGACATGATCA		
*gyr*A	F-CGCATGRCCAAGCTGGC	*P. stutzeri*	This study
	R-TAGTTGGGCACCCAGTCG		
*kat*B	F-CGGCTTCGCCACCAAGTT	*P. stutzeri*	This study
	R-GTCGGGAAGTTGTTGCCGA		

Total RNA was extracted from *P. stutzeri* in samples that were either untreated or treated by 3 h of incubation with 5 g L^−1^ of nZVI using the Real Total RNA Spin Plus Kit (Durviz S.L., Valencia, ES). Reverse transcription (RT) for each target mRNA was performed according to the iScriptTM Select cDNA Synthesis Kit manufacturer’s instructions using the specific reverse primers (Table 1) and the protocol previously described [1]. The reported expression levels of the target genes were quantified in reference to those of the internal 16S rDNA reference gene. Standard curves were generated for each of the target genes analysed. Expression ratios that exhibited a 3-fold or more change (above or below) were considered to be significant.

### 5. Proteomic Analysis

Cells incubated in 250 mL of LB broth (37°C, 120 rpm) were collected at the exponential growth phase and treated with 5 g L^−1^ nZVI for 3 h to allow for protein expression in response to nZVI. The cells were then pelleted by centrifugation (5000 rpm, 10 min, 4°C) and washed twice with Tris-HCl 0.1 M buffer (pH 7.5). They were resuspended in 5 mL of the same buffer, and then, 500 µL of a protease inhibitor solution (SIGMA P2714) was added. The cells were then subjected to 4 repeated cycles of agitation (5 m s^−1^, 15 s in a FastPrepTM machine) and cooled on ice. After centrifugation (6400 rpm, 4°C, 15 min), the supernatant lysates were precipitated with 10% trichloroacetic acid and acetone (stored at −20°C) and resuspended in 7 M urea, 2 M thiourea, and 4% (w/v) CHAPS.

The entire proteomes from control and nZVI treated *P. stutzeri* were compared using two-dimensional difference gel electrophoresis (2D-DIGE) to identify proteins with differential expression in response to nZVI stress. Four biological replicates were used from the control and treated samples. The experimental design included a Cy2-labelled internal standard containing an equal amount of protein from all of the replicates. A dye swap was performed with Cy3 and Cy5 dyes to ensure that the differences between the two conditions were not due to differences in the dye labelling. In total, 50 µg of each sample was fluorescently labelled by following the CyDyes minimal manufacturer’s instructions (GE Healthcare).

The labelled proteins were mixed according to the experimental design and applied to Immobilize pH gradient (IPG) strips (24 cm in length, 4–7 pH range) that had been hydrated overnight. Isoelectric focusing (IEF) was performed at 20°C using the following program: 120 V for 1 h, 500 V for 1 h, 500–1000 V for 2 h, 1000–4500 V for 6 h, and 4500 V for 12 h. Subsequently, the strips were equilibrated in a reducing solution [6 M urea, 50 mM Tris-HCl at pH 6.8, 30% (v/v) glycerol, 2% (w/v) SDS, and 2% (w/v) DTT] followed by an alkylating solution [6 M urea, 50 mM Tris-HCl at pH 6.8, 30% (v/v) glycerol, 2% (w/v) SDS, and 2.5% (w/v) iodoacetamide]. A 2D SDS-PAGE was run on homogeneous 12% T and 2.6% C polyacrylamide gels cast in low-fluorescent glass plates. Electrophoresis was carried out at 20°C, 2 W/gel for 18 h, using an Ettan-Dalt six unit. The gels were scanned immediately after SDS-PAGE using a Typhoon 9400 scanner (GE Healthcare) with photomultiplier tubes that were optimised for each laser to achieve the broadest range. Images were scanned at a resolution of 100 µm.

The fluorescent gel images were cropped using ImageQuant v5.1 software (GE Healthcare) and analysed using DeCyder v6.5 software (GE Healthcare). The differential in-gel analysis (DIA) module was used to assign spot boundaries and to calculate parameters such as normalised spot volumes. The internal variability was corrected by matching and normalising with the internal standard spot maps in the biological variation analysis (BVA) module. The average fluorescence ratio was calculated for control, and treated samples and unpaired Student’s t test was performed to determine significance. Protein spots with an average ratio higher than 1.5 and a p value <0.05 were considered to be significant and differentially expressed between the groups.

After fluorescence scanning, the 2D gels were stained with colloidal coomassie blue. Protein identification was performed at the Proteomic Facility from the Universidad Complutense de Madrid-Fundación Parque Científico de Madrid (UCM-FPCM), Spain, a member of the ProteoRed-ISCIII Network, and the results are described in File S1.

## Results and Discussion

### 1. Cellular Viability and TEM Analysis

**Figure 1:**
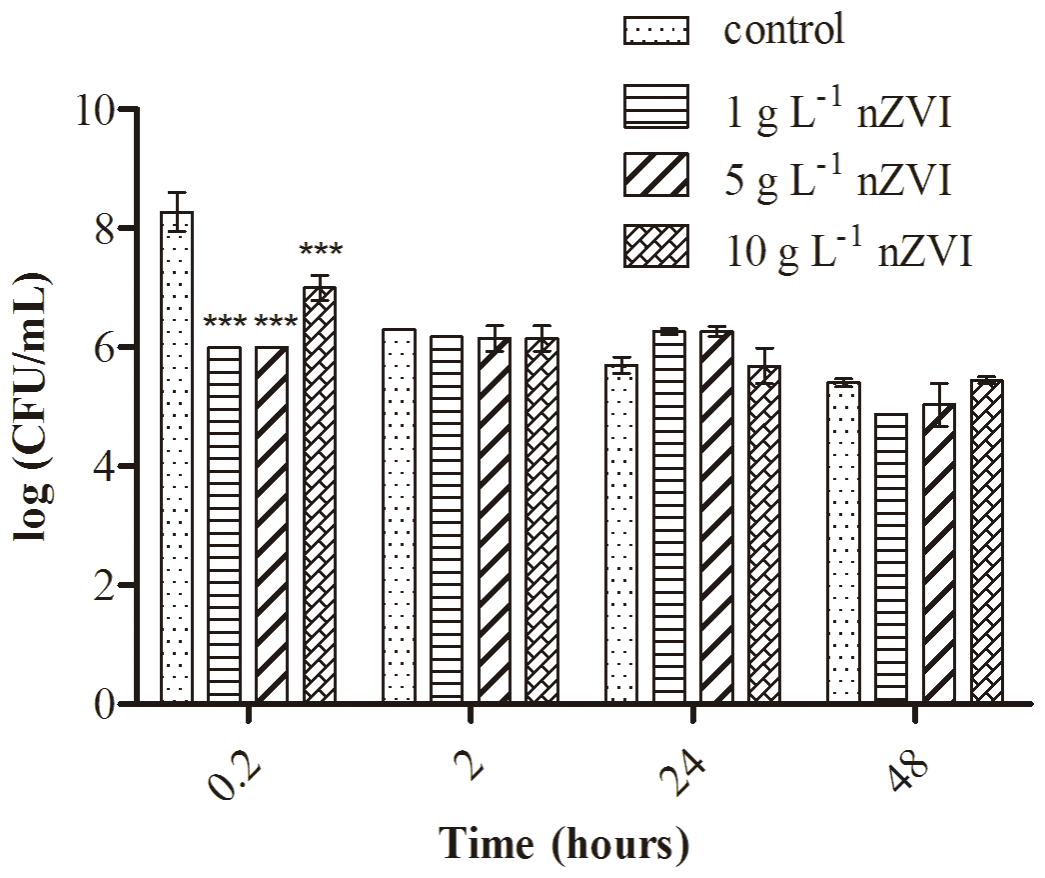
Cell viability of the Gram negative strain *P. stutzeri* after exposure to nZVI particles.

nZVI cytotoxicity on *P. stutzeri* was assessed using a liquid-to-plate assay in which nanoparticle-bacterial interactions can be observed in the absence of any medium effect. Fig. 1 shows the cell viability of this Gram negative strain when treated with three concentrations of nZVI over different exposure times. nZVI concentrations used were in the range of those employed in previous sites for environmental remediation [1], [2], [9]. In contrast to previous studies carried out with *P. fluorescens* [10] and *E. coli* [11], which demonstrated a strong cellular inactivation in bacteria following nZVI treatment, *P. stutzeri* exhibited a higher resistance. Similar results were found in a previous study on the impact of nZVI on the Gram negative bacteria, *Klebsiella* sp. [1], [12]. Here, we report that nZVI particles were only toxic to *P. stutzeri* at doses ranging from 1–5 g L^−1^ and only within the first 10 min of incubation. Because nanoparticle aggregation is an important toxicity-determining parameter, this result could be explained by the fact that higher concentrations may produce a higher aggregation of nZVI particles, thus reducing their reactivity and their antimicrobial effect. Moreover, increasing exposure times would lead to increased oxidation and passivation of nanoparticles, ultimately reducing their reactivity.

Therefore, considering results from the viability assay, an intermediate subtoxic nZVI concentration (5 g L^−1^) was used for further analyses.

**Figure 2:**
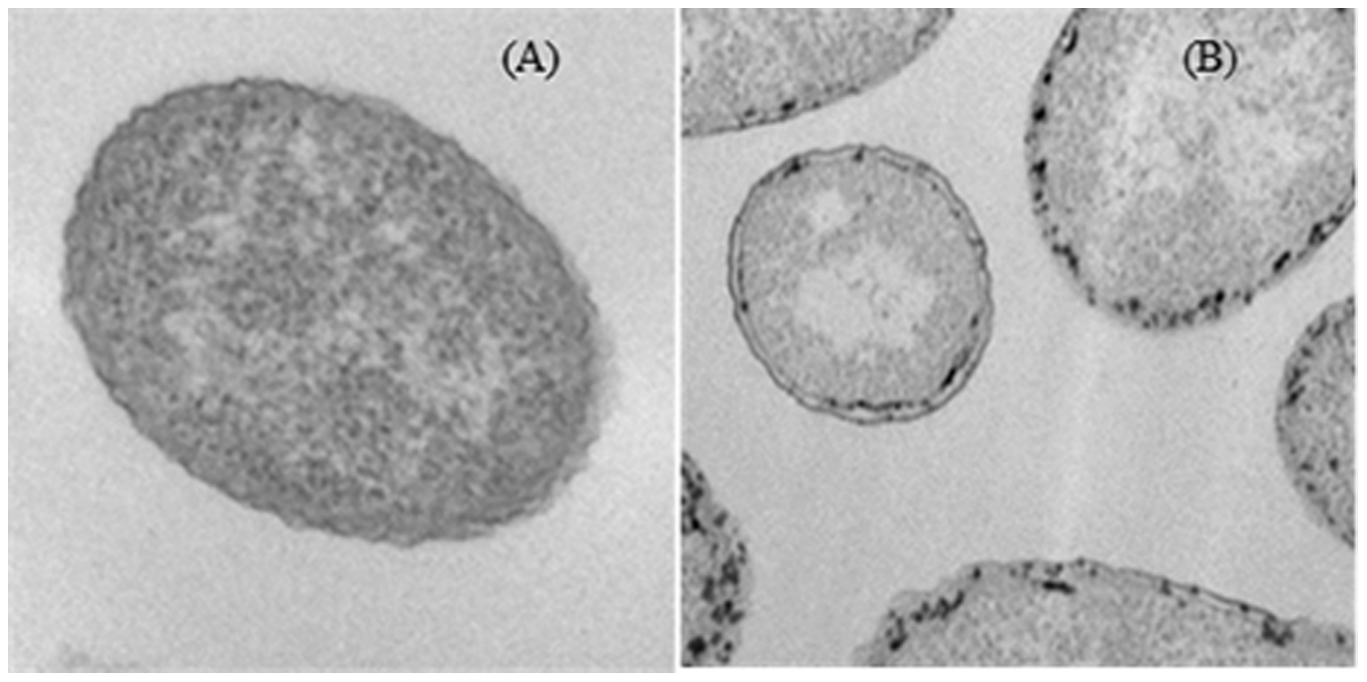
TEM micrographs showing *P. stutzeri* control cells (A) and cells exposed to 5 g L^−1^ nZVI (B).

The investigation of nZVI effects on cell morphology by TEM revealed interesting findings (Fig. 2). In the absence of nanoparticles, TEM images showed a well-defined cell wall and an evenly stained cell interior (Fig. 2A). In nZVI-treated bacteria (Fig. 2B), nanoparticle attachment to the cell membrane was observed (in approximately 50% of treated cells), although they were not observed in the cytoplasm. In previous works [13] the prokaryotic cell wall has been described as a barrier to nanoparticles that can strongly influence nanoparticle-cell interactions; however, the mechanisms underlying such interactions have not been fully investigated. The results from the TEM micrographs suggest that the initial step of nZVI toxicity involves an insult to the bacterial cell wall. The cell wall of *P. stutzeri* likely acts as a barrier to prevent nZVI entry into the cytoplasm, suggesting a key role for membrane proteins in the cellular toxicity response.

### 2. Transcriptional Analyses

When studying the biological effects of nanoparticles it is necessary to consider the temporal shift between the changes produced by stressors at the cellular and molecular level, and the corresponding alterations at the phenotypic level.

We describe the transcriptional response of *P. stutzeri* to nZVI treatment using five genes of particular relevance in cellular activity (*narG, nirS, pykA, gyrA* and *katB*). The expression level of the target genes in comparison to the reference gene (16S rRNA) was determined by RT-qPCR (Table 2). Exposing *P. stutzeri* to nZVI did not significantly change the expression ratio of *nar*G, *nir*S, *pyk*A and *gyr*A genes, whereas *kat*B expression was significantly increased (5.7-fold higher than the control). Catalase is an enzyme that is actively involved in cellular oxidative stress detoxification. Nanoparticles, in particular metal nanoparticles, can generate ROS, which in turn, can trigger the oxidative stress response. The upregulation of the gene *kat*B following nZVI exposure suggests that *P. stutzeri* actively responds to induced oxidative stress. Considering the mild effect of nZVI on *P. stutzeri* viability, the cellular response might be involved in minimising any further acute toxic effects.

**Table 2:** Gene expression fold change in *P. stutzeri* cells treated with 5 g L^−1^ nZVI.

Target gene	Gene expression fold change
*nar*G	1.18
*nir*S	1.01
*pyk*A	2.55
*gyr*A	1.40
*kat*B	5.72

### 3. Proteomic Assay

**Figure 3:**
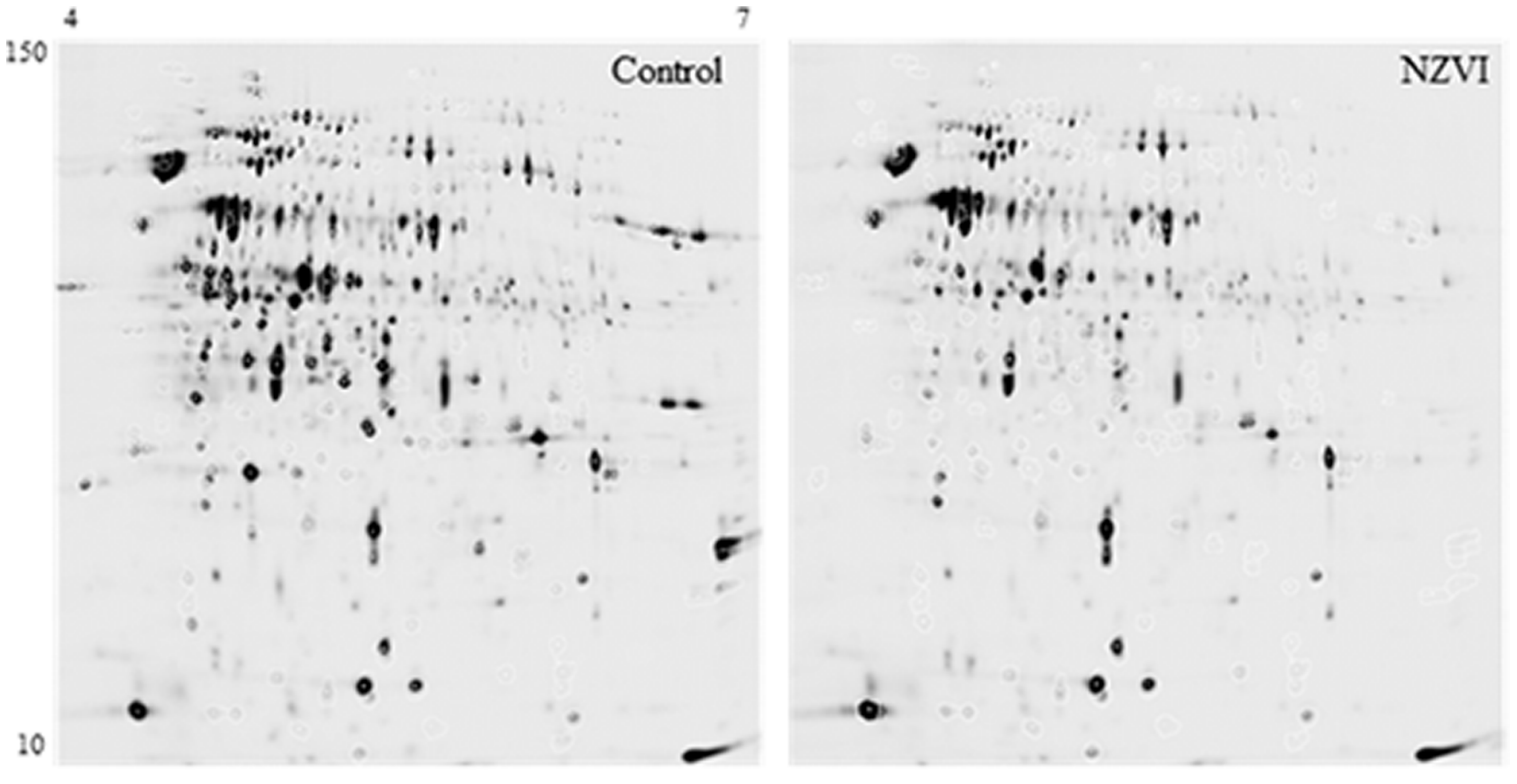
Protein profiles of control and nZVI-treated bacteria are shown in the gels. Proteins excised from the gels for identification have been highlighted in white.

DIGE revealed that 190 spots exhibited significant changes in protein abundance as a consequence of nZVI exposure. Using these 2D profiling data, a principal component analysis (PCA) distinguished between the control and nZVI-exposed bacteria (Fig. S1, S2). Among the protein spots, the 28 most relevant were excised from the 2D GE gels for identification and quantification (Fig. 3). Among these, 18 were downregulated and 10 were upregulated as a consequence of nZVI exposure. The most relevant response of *P. stutzeri* to nZVI-induced stress was the modulation of both membrane proteins (downregulated) and proteins involved in the defence against oxidative stress (upregulated).

The downregulated proteins were grouped according to their biochemical functionality: membrane proteins, metabolic enzymes, and others (Table 3).

**Table 3:** Differentially expressed proteins identified in *P. stutzeri* specifically downregulated by nZVI treatment.

Accession n°	Protein name and/or locus tag	Functional classification	Ratio
gi|379064074	ribonucleotide-diphosphate reductase subunit alpha (*nrd*A)	Metabolism: nucleotides	−13.5
gi|379066251	inosine 5′-monophosphate dehydrogenase (*gua*B)	Metabolism: nucleotides	−2.37
gi|379066031	bifunctional aconitate hydratase 2/2-methylisocitrate dehydratase (*acn*B)	Metabolism: TCA pathway	−5.89
gi|333898825	translation elongation factor Tu (*tuf-1*)	Metabolism: Protein synthesis	−4.96
gi|128515	nitrous-oxide reductase (*nos*Z)	Metabolism: nitrogen cycle	−7.59
gi|128346	nitrite reductase (*nir*S)	Metabolism: nitrogen cycle	−13.9
gi|379063430	outer membrane protein OprE3 (*opr*Q)	Transporter membrane protein	−26.9
gi|379066257	outer membrane protein OprC (*opr*C)	Transporter membrane protein	−2.53
gi|379065307	outer membrane protein H1 (*op*rH)	Transporter membrane protein	−31.67
gi|379066122	TonB-dependent siderophore receptor (PST_0996)	Transporter membrane protein	−32.57
gi|379063710	Porin (PstZobell_03306)	Transporter membrane protein	−17.25
gi|379064573	putrescine ABC transporter periplasmic putrescine-binding protein (*pot*F)	Transporters: ABC-type	−9.34
gi|379065016	iron ABC transporter periplasmic iron-binding protein (PST_4066)	Transporters: ABC-type	−3.34
gi|379064032	phosphonate ABC transporter periplasmic phosphonate-binding protein(PST_2977)	Transporters: ABC-type	−6.67
gi|379064616	C4 dicarboxylate binding protein (PST_1720)	Transporter membrane protein	−13.21
gi|379063134	periplasmic protein (PstZobell_00382)	Transporter membrane protein	−23.27
gi|327482149	periplasmic protein (PSTAA_3599)	Transporter membrane protein	−17.7
gi|379066790	hypothetical protein, partial (PstZobell_19118)	Others	−25.39

Membrane proteins are involved in a variety of essential cellular processes, such as cell signalling, material transport and the stress response. Damage to cell wall components can affect the cellular mechanical defences, allowing for the passage of nanoparticles across the cell membrane, which in turn can cause serious cellular damage [14]–[16]. Therefore, damage to the microbial cell envelope could serve as a basis for new ecotoxicity assays involving nanomaterials [13]. In this study, the abundance of several membrane proteins was significantly reduced following nZVI exposure. Among them, 8 protein spots that were dramatically affected by nZVI treatment (Table 3) were identified as porins and transporters. ABC transporters play important roles in nutrient uptake and the export of toxic substances. Interestingly, we found that the iron ABC transporter periplasmic protein, PST 4066 and TonB-dependent siderophore receptor (see Table 3), both which are involved in iron uptake, were downregulated. An abundance of iron promotes the formation of ROS, in turn leading to the damage of key cellular components, and thus, the decreased abundance of both proteins should diminish the availability of iron within the cell.

Proteins involved in the nitrogen cycle including cytochrome cd1-containing nitrite reductase and nitrous-oxide reductase (encoded by *nir*S and *nos*Z genes, respectively) were downregulated following nZVI exposure. Both are membrane-associated proteins and, therefore, are susceptible to damage and/or degradation by environmental stressors such as nZVI. However, the decrease in the NirS protein levels did not correspond to the decrease in *nir*S gene transcription level. This fact can be explained considering that post-translational modifications and/or degradation of proteins can result in differences between the actual proteome and that predicted by transcriptome analysis [7].

Two proteins identified as metabolic enzymes involved in providing precursors for DNA and/or RNA biosynthesis including ribonucleotide-diphosphate reductase subunit alpha (RNR) and inosine 5′-monophosphate dehydrogenase (IMPDH) were downregulated following nZVI exposure. Both oxidoreductase enzymes could be affected by an oxidising cellular environment created by nZVI exposure [17]. Moreover, IMPDH catalyses the first rate-limiting step in GTP synthesis [18]. The downregulation of this protein was coupled to a decrease in translation elongation factor Tu (EF-Tu) abundance, which may suggest that nZVI-induced stress results in impaired protein synthesis.

Mailloux et al. [19] determined an intricate link between the tricarboxylic acid (TCA) cycle and ROS formation. Thereby, decreased TCA cycle activity during an oxidative insult could result in diminished levels of NADH and lower ROS production via the electron transport chain. This would explain the downregulation of aconitate hydratase, an enzyme involved in the TCA cycle with an active [Fe_4_S_4_]^2+^ cluster that is highly sensitive to ROS.

In this study, several upregulated proteins grouped as metabolic, oxidative, or general stress responsive proteins were identified (Table 4).

**Table 4:** Differentially expressed proteins identified in *P. stutzeri* specifically upregulated by nZVI treatment.

Accession n°	Protein name and/or locus tag	Functional classification	Ratio
gi|379065454	delta-aminolevulinic acid dehydratase (*hem*B)	Metabolism: tetrapyrrole biosynthesis	+2.14
gi|379066374	50S ribosomal protein L25 (PstZobell_16978)	General stress protein; protein metabolism	+1.99
gi|379065351	malate synthase G homologue (**glc** ***G***)	Metabolism: carbohydrates	+2.3
gi|15600481	glnK gene product (*gln*K)	Metabolism: nitrogen regulatory protein	+2.16
gi|379063228	acetyl-CoA carboxylase biotin carboxyl carrier protein subunit (*acc*B)	Metabolism: lipids. Fatty acids biosynthesis	+2.27
gi|302189738	co-chaperonin GroES (Psyrps6_010100025479)	Protein destination and storage: folding and stability	+2.04
gi|3913226	chaperonin GroES protein (*gro*ES)	Protein destination and storage: folding and stability	+2.1
gi|379065998	chaperonin GroEL (*gro*EL)	Protein destination and storage: folding and stability	+2.24
gi|379063758	heat shock protein GrpE (*grp*E)	Protein destination and storage: folding and stability	+2.7
gi|15599562	*sod*B gene product (*sod*B)	Antioxidants, detoxification	+2.12

Delta-aminolevulinic acid dehydratase (ALAD), encoded by the *hem*B gene, was significantly upregulated. This enzyme converts two δ-ALA molecules into a monopyrole porphobilinogen; this is the first common step in tetrapyrrole synthesis and the major point of regulation in the tetrapyrrole biosynthesis pathway [20]. Catalysis and structure of the ALAD enzyme require metals, in particular iron has a positive effect on this protein’s level and activity, for its crucial importance in heme biosynthesis. The heme group is the prosthetic group of several enzymes involved in different cellular processes, such as heme catalase [21]. This enzyme catalyses the disproportionate reaction of hydrogen peroxide and is directly involved in protecting against cellular oxidative stress. Moreover, the heme group can act as an iron scavenger under the cytoplasmatic iron-replete conditions. Thus, the increased abundance of ALAD suggests an increase in the biosynthesis of the heme group after cells are treated with nZVI particles. This would likely result in diminished harmful ROS effects and scavenge the excess of iron.

In this study, the main classes of upregulated proteins following nZVI exposure were general stress and oxidative stress responsive proteins (Table 4); chaperonins and heat shock proteins were identified in the former group. Chaperonins provide favourable conditions for the correct folding of other proteins to prevent their aggregation. Members of the GroEL/GroES complex are among the best characterised bacterial chaperonins. This complex is responsible for preventing protein damage in response to high levels of stress. Heat shock proteins (HSPs) function as chaperones, which facilitate in stabilising partially unfolded proteins and are well known to be upregulated when cells are exposed to several stressors [22]. Our results indicate a similar role for these proteins following nZVI treatment. Moreover, 50S ribosomal protein L25 was also upregulated (Table 4). L25 in many bacteria shows significant homology to the N-terminal domain of the *B. subtilis* Ctc protein, which has been described as a general stress protein [23]. We hypothesise that a similar scenario, such as the upregulation of the expression of this stress responsive protein, may occur in *P. stutzeri* following nZVI exposure.

Fe-superoxide dismutase (SodB), which acts as superoxide scavenger was also found to be upregulated. Similar results have been reported in *P. stutzeri* following quantum dots exposure [24]. Superoxide dismutases (Sods) are ubiquitous enzymes found in nearly all organisms and they play a major role in the multi-defence system against oxidative stress [25]. Two Sod enzymes have been identified in bacteria: a manganese Sod (MnSod) and an iron Sod (FeSod), encoded by *sod*A and *sod*B, respectively. Oxidative stress is potentiated by iron because it reacts with H_2_O_2_ in the Fenton reaction. There is increasing evidence of the coordination between the regulation of iron homeostasis and the defences against oxidative stress [26]. Remarkably, SodB is produced exclusively in high iron growth conditions [27]. In our study, the upregulation of SodB under conditions of iron repletion due to nZVI exposure provides evidence for the coordination between the regulation of iron homeostasis and defences against oxidative stress in *P. stutzeri*.

## Conclusions

This study describes the cellular response of *P. stutzeri* to nZVI exposure. The phenotypic response indicated that the cell membrane was affected by nanoparticle attachment on the bacterial wall. The transcriptomic analysis suggested that nZVI exposure induced oxidative stress in *P. stutzeri,* revealed by the upregulation of *kat*B gene expression. The proteomic approach provided new insights to confirm these previous observations. Changes in the protein expression of *P. stutzeri* indicated that the cell employs a molecular response to counteract the impact of nZVI particles through two major defence mechanisms: the repression of membrane proteins to control iron uptake (iron ABC transporter periplasmic protein and TonB-dependent siderophore receptor) and the overproduction of proteins to scavenge ROS, thereby reducing intracellular oxidative stress and preventing abnormally folded proteins (chaperonins, heat shock proteins and superoxide dismutase).

The molecular approach outlined in this work elucidates novel, relevant proteins that could be utilised as biomarkers of toxicity, such as catalase and superoxide dismutase, in addition to the standard ecotoxicological tests. Long-term exposure and more complex media must also be considered to improve our understanding of the impact of nZVI particles on environmental biota in realistic scenarios.

## Supporting Information

Figure S1 **PCA clustered the 8 individual images from Cy3- and Cy5-labeled dyes samples, using the data from the differentially expressed protein spots into two groups.**
(TIF)

Figure S2 **Hierarchical cluster analysis of the differentially expressed proteins, using Euclidean distance measurements and average linkage.** The dendrogram of eight individual image clustering is shown at the top, and that of individual proteins is shown on the right, with relative expression values being displayed in a heat map.
(TIF)

File S1 **Detailed Materials and Methods describing the identification of protein spots by MALDI-TOF MS.**
(DOC)
