# Supplementary figures and images for: Molecular Stress Responses to Nano-Sized Zero-Valent Iron (nZVI) Particles in the Soil Bacterium Pseudomonas stutzeri
Source: PLoS One. 2014 Feb 25;9(2):e89677. doi: 10.1371/journal.pone.0089677 (PMC3934913; doi:10.1371/journal.pone.0089677)

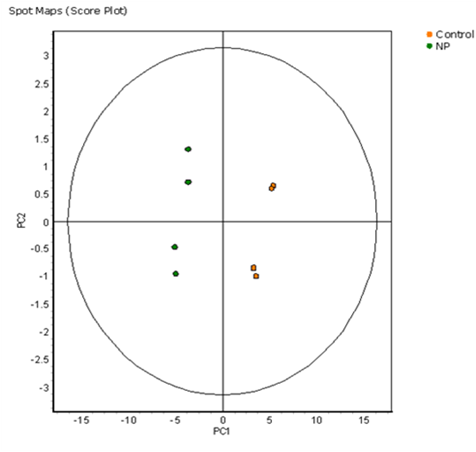

Supplement: Figure S1 — PCA clustered the 8 individual images from Cy3- and Cy5-labeled dyes samples, using the data from the differentially expressed protein spots into two groups. (TIF) [file pone.0089677.s001.tif]

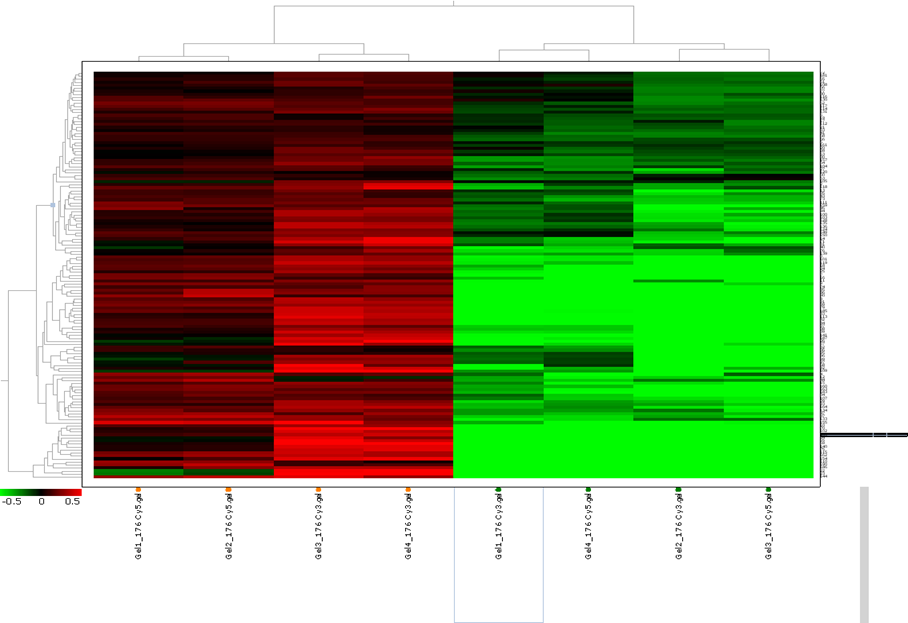

Supplement: Figure S2 — Hierarchical cluster analysis of the differentially expressed proteins, using Euclidean distance measurements and average linkage. The dendrogram of eight individual image clustering is shown at the top, and that of individual proteins is shown on the right, with relative expression values being displayed in a heat map. (TIF) [file pone.0089677.s002.tif]
