# Supplementary material for: Molecular Stress Responses to Nano-Sized Zero-Valent Iron (nZVI) Particles in the Soil Bacterium Pseudomonas stutzeri
Source: PLoS One. 2014 Feb 25;9(2):e89677. doi: 10.1371/journal.pone.0089677 (PMC3934913; doi:10.1371/journal.pone.0089677)
Supplement: File S1 — Detailed Materials and Methods describing the identification of protein spots by MALDI-TOF MS. (DOC) [file pone.0089677.s003.doc]

After fluorescence scanning, the 2-DE gels were stained following the colloidal coomassie blue protocol. Spots selected for analysis were in-gel reduced, alkylated and digested with trypsin. After digestion, the supernatants were collected, and 1 μl was spotted onto a matrix-assisted laser desorption ionization (MALDI) target plate and allowed to air-dry at room temperature. Then, 0.5 µl of a 3 mg/ml of α-cyano-4-hydroxy-cinnamic acid matrix in 0.1% TFA-50% ACN was added to the dried peptide digest spots and again allowed to air-dry. Samples were analyzed using MALDI-TOF/TOF mass spectrometer 4800 plus Proteomics Analyzer (Applied Biosystems. MDS Sciex, Toronto, Canada) and 4000 Series Explorer™v 3.5 Software (ABSciex). MS spectra were acquired in reflector positive-ion mode using 1000 laser shots per spectrum. Mass spectra were internally calibrated using autoproteolytic trypsin ions. MS/MS fragmentation spectra were acquired by selecting some of the most abundant ions up to 10 of each MALDI-TOF peptide mass spectra (excluding trypsin peptides and other known background ions) and averaging up to 3000 laser shots per fragmentation spectrum. Fragmentation was carried out using the operating method 1 kV ion reflector mode with CID on (atmospheric gas used) and precursor mass window ± 4 Da. GPS explorer v 3.5 (ABSciex) software was used for the spectra analyses and to generate peaking lists. The monoisotopic peptide mass fingerprinting data obtained from MS and the mass of the fragments obtained from each peptide fragmentation in MS/MS analyses were used to search for protein candidates using Mascot v. 2.2 from Matrix Science (http://www.matrixscience.com). Peak intensity was used to select 65 peaks per spot for peptide mass fingerprinting, and 65 peaks per precursor for MS/MS identification. Tryptic autolytic fragments, keratin and matrix derived peaks were removed from the dataset used for the database search. The searches for peptide mass fingerprints, tandem MS spectra and these two combined were performed in the NCBInr database (date: 17919084 sequences and 6150218869 residues) without taxonomy restriction. The Mascot search parameters were (1) species, all; (2) allowed number of missed cleavages, 1; (3) fixed modification, carbamidomethyl cystein; (4) variable modifications, methionine oxidation; (5) peptide tolerance, ±50-100 ppm; (6) MS/MS tolerance, ±0.3 Da; and (7) peptide charge, +1. In all of the identified proteins, the probability score was greater than the one fixed by Mascot as being significant, that is, a p value less than 0.05.
